# IL-27 Modulates the Cytokine Secretion in the T Cell–Osteoclast Crosstalk During HIV Infection

**DOI:** 10.3389/fimmu.2022.818677

**Published:** 2022-04-05

**Authors:** Tong Li, Colleen Hadigan, Jarred M. Whitlock, Jing Qin, Jai Kumar, Princy Kumar, Marta Catalfamo

**Affiliations:** ^1^ Department of Microbiology and Immunology, Georgetown University School of Medicine, Washington, DC, United States; ^2^ Laboratory of Immunoregulation, National Institute of Allergy and Infectious Diseases, National Institutes of Health, Bethesda, MD, United States; ^3^ Section on Membrane Biology, Eunice Kennedy Shriver National Institute of Child Health and Human Development, National Institutes of Health, Bethesda, MD, United States; ^4^ Biostatistics Research Branch, Division of Clinical Research (DCR), National Institutes of Allergy and Infectious Diseases (NIAID), National Institutes of Health (NIH), Bethesda, MD, United States; ^5^ Division of Infectious Diseases and Travel Medicine, Georgetown University School of Medicine, Washington, DC, United States

**Keywords:** IL27, HIV, T cell immune activation, Th1, T cell:osteoclast

## Abstract

In People with HIV (PWH), chronic immune activation and systemic inflammation are associated with increased risk to develop comorbidities including bone loss. Numerous cells of the immune system, namely, T cells are involved in the regulation of the bone homeostasis and osteoclasts (OCs) activity. IL-27, a cytokine that belongs to the IL-12 family can regulate the secretion of pro- and anti-inflammatory cytokines by T cells, however its role in the setting of HIV is largely unknown. In the present study, we determined the impact of OCs in T cell secretion of cytokines and whether IL-27 can regulate this function. We found that the presence of OCs in the T cell cultures significantly enhanced secretion of IFNγ, TNFα, IL-17, RANKL, and IL-10 in both PWH and healthy controls. In PWH, IL-27 inhibited IL-17 secretion and downregulated surface expression of RANKL in CD4 T cells. All together these results suggest that in the context of HIV infection IL-27 may favor IFNγ and TNFα secretion at the sites of bone remodeling.

## Introduction

In People with HIV (PWH), chronic immune activation and systemic inflammation are associated with an accelerated course of chronic diseases including osteoporosis, an important contributor of morbidity and mortality in these patients (1–5). Low bone mineral density (BMD) is multifactorial and represents a complex interplay between HIV infection, traditional risk factors of osteoporosis, and effects of the antiretroviral therapy. Reports have shown that HIV infection is a risk factor for low BMD in young (6–11) and older individuals (12–16). The molecular mechanisms linking HIV infection and bone disease are largely unknown.

In the bone, there is a coordinated balance between bone-forming osteoblast cells, and bone-resorbing osteoclasts (OCs) cells. OC precursors are monocyte/macrophage lineage and are recruited from the bone marrow and circulation into the sites of bone remodeling (17–23). During bone remodeling, OCs precursors undergo differentiation through signals provided by the macrophage colony-stimulating factor (M-CSF) and the receptor of activation of nuclear factor kappa B (NF-κB) (RANK) ligand (RANKL) (24). Osteoblasts were thought to be the main sources of RANKL promoting the differentiation of OCs precursors, however new evidence has determined that osteocytes produce RANKL and therefore are important players in regulating adult bone remodeling (25–28). The availability of RANKL in the microenvironment of the bone is regulated by Osteoprotegerin (OPG), which acts as a decoy receptor restricting osteoclastogenesis (17, 29–32).

Regulation of the bone homeostasis is modulated by cells of the immune system (33). B cells and T cells secrete RANKL and OPG, key factors involved in bone remodeling (34–38). Particularly, T cells are sources of RANKL and other cytokines, namely, IFNγ, TNFα, and IL-17 regulating osteoclasts differentiation and bone resorption (39). IL-17 synergizes with RANK–RANKL pathway promoting OCs activity (33, 36, 40–43). In contrast, IFNγ has dual effects by interfering with OCs differentiation; and indirectly IFNγ enhances OCs antigen presentation capacity promoting T cell activation and consequently the T cell secretion of pro-osteoclastogenic cytokines, including, RANKL and TNFα (44–47). In addition, IL-10 a regulatory cytokine has inhibitory effects both direct and indirect in osteoclastogenesis (48–51). In T cells, IL-10 has wide regulatory functions and inhibit production of pro-inflammatory cytokines (52).

In the setting of HIV infection, the role of T cell immune activation in bone homeostasis is not totally understood. Reports have shown that T cell reconstitution and immune activation is associated with bone loss (53–56). In PBMCs (Peripheral Blood Mononuclear Cells) from PWH, both CD4 and CD8 T cells express higher RANKL and decreased OPG, and its ratio is correlated with BMD suggesting a potential contribution of T cells in bone loss (53, 54, 57, 58).

IL-27 a cytokine that belongs to the IL-12 family has been shown to play a role in the bone remodeling (59). IL-27 is a heterodimer formed by the IL-27p28 chains and Epstein–Barr Virus-induced gene 3 (Ebi3) chain (60–63). IL-27 signals through a heterodimer receptor composed of IL-27Rα and gp130 and activates Janus kinase (JAK)-signal transducer and activator of transcription (STAT-1 and -3), and the mitogen activated protein kinase (MAPK) pathway (60, 63, 64). IL-27 plays a crucial role in immunity, balancing protective and inflammatory responses including development of helper T (Th)1 and inhibition of Th17 and Th2 differentiation (61, 65–67). In addition, IL-27 induces upregulation of PD-L1 expression, and generation of IL-10-producing type 1 regulatory T (Tr1) cells (64, 67–75).

In the bone, recent evidence has shown that IL-27 is expressed in both osteoblasts and osteoclasts and can directly modulate bone turnover by downregulating RANK expression (59, 76–78). In addition, in CD4 T cells, IL-27 inhibits RANKL secretion modulating their pro-osteoclastogenic function and suppressing T cell-mediated inflammatory bone destruction (79).

The impact of HIV driven T cell immune activation in the T cell–osteoclast (T:OCs) crosstalk and whether IL-27 can modulate T cell function in this setting is not well defined. In the present study, we developed an *in vitro* coculture system and investigated the cytokine networks involved, and their regulation by IL-27. We found that OCs significantly increased the secretion of cytokines (IL-17, TNFα, IFNγ, RANKL, and IL-10) by T cells from healthy controls and PWH. In T cells from PWH, IL-27 downregulated IL-17 and RANKL surface expression, suggesting that in the setting of HIV infection, IL-27 may favor a Th1 associated cytokines at the site of bone remodeling.

## Material and Methods

### Study Participants

Participants were studied under a MedStar Georgetown University Hospital and an NIAID Institutional Review Board approved HIV clinical research studies. Characteristics of the study groups of PWH (n = 26) are described in **Table 1**. Healthy volunteers (n = 19) were obtained from the MedStar Georgetown University Hospital, and the NIH Blood Bank under an institutional review board approved protocol (**Table 2**). All study participants signed a written informed consent for the collection of samples.

**Table 1:** Characteristic of the study participants.

	HIV^+^(n = 9)^a^	HIV^+^ (T)(n = 17)
**Age, yr, median (IQR)**	52.0 (44.5–57.5)	48 (42–53)
**Gender n (%)**		
Male	7 (77.8)	14 (82.4)
Female	2 (22.2)	3 (17.6)
**Race/Ethnicity n (%)**		
White	3 (33.3)	6 (35.3)
Black	5 (55.6)	9 (52.9)
Other	1 (11.1)	2 (11.8)
Years on ARV median (IQR)	N/A	4.692 (3.221–9.166)
**Clinical Characteristics**		
Viral load (copies/ml, IQR)	<20 (20–20)^b^	<50^c^
ASCVD risk median (IQR)	4.6 (3.4–20.2)	N/A
FRS median (IQR)	N/A	3.1 (2.2–6.5)^d^
Total Mass kg median (IQR)	N/A	80.70 (66.70–87.85)
Total BMD g/cm^2^ median (IQR)	N/A	1.165 (1.057–1.330)^e^
Spine BMD/cm^2^ median (IQR)	N/A	1.109 (0.939–1.230)
T-score median (IQR)	N/A	−0.30 (−0.95–1.55)
BMI median (IQR)	N/A	27.10 (23.06–29.80)
Metabolic syndrome n (%)	N/A	6 (35.3)
DBP mmHg median (IQR)	85 (71–92)	76.0 (71.0–80.5)
**T cell counts median (IQR)**		
CD4 counts (cells/μl)	524.0 (251.0–833.5)	525.0 (449.5–689.0)
CD8 counts (cells/μl)	N/A	718.0 (571.0–1227.0)
Nadir CD4	N/A	241.0 (90.0–350.0)
**Clinical Laboratory, median (IQR)**		
Total Cholesterol mg/dl	168 (152–207)	170.0 (134.5–196.0)
LDL mg/dl	76 (53–125)	96.00 (47.25–114.00) ^e^
HDL mg/dl	67 (51–84)	45.0 (34.5–58.5)
Triglycerides	N/A	118 (100–182)
D-Dimer	N/A	0.270 (0.225–0.465)
CRP	N/A	1.44 (0.77–5.01)

a Total cholesterol, LDL and HDL, ASCVD data are not available in 6 out 9 participants.

b 1 out of 9 had VL of 28 copies/ml.

c 3 out 17 participants had VL >50 copies/ml.

d FRS. Not available in 2 donors.

e Total BMD and LDL are not available in 1 out 17 participants.

ARV, Antiretrovirals; ASCVD, Atherosclerotic Cardiovascular Disease risk; FRS, Framingham Risk Score; BMD, Bone Mineral Density; BMI, Body Mass Index; DBP, Diastolic Blood Pressure; LDL, Low-Density Lipid; HDL, High-Density Lipid; CRP, C Reactive Protein; IQR, Interquartile range. N/A, Not Available.

**Table 2:** Characteristic of the healthy controls.

Healthy controls	HC (n = 10)^a^ Figure 1	HC (n = 9)^b^ Figure 2
**Age, yr, median (IQR)**	53 (38–60)	34 (29–43)
**Gender n (%)**		
Male	4 (40)	5 (55.6)
Female	5 (50)	2 (22.2)
**Race/Ethnicity n (%)**		
White	5 (50)	4 (44.4)
Black	3 (30)	N/A
Other	1 (10)	3 (33.3)

a Age, gender and race data are not available in 1 out of 10 participants.

b Age, gender and race data are not available in 2 out of 9 participants. N/A, Not Available.

### Tissue Culture

#### Osteoclasts Differentiation

Osteoclasts were differentiated from frozen PBMCs as described (80). Briefly, frozen PBMCs from healthy donors (HC, n = 19) and PWH (n = 26) were thawed and 1 × 10^6^ cells were cultured in a 12-well plate in conditioned media MEM-α (Gibco, MA) containing 10% heat-inactivated FBS (Gemini, CA), and M-CSF (20 ng/ml, R&D System, MN) at 37°C and 5% CO_2_. Cells were fed with fresh media every three days. After 6 to 9 days of culture, osteoclast precursors were differentiated by the addition of RANKL (20 ng/ml, R&D System, MN) to the media (MEM-α containing M-CSF (20 ng/ml, R&D System, MN)). At day 6, osteoclast differentiation was determined by microscopy and measuring tartrate-resistance acid phosphatase (TRAP) activity as described (80, 81).

#### Microscopy

Cells were cultured in coverslips and fixed with 4% paraformaldehyde for 15 min and washed with PBS. Hoechst 33342 (Invitrogen, MA) and Alexa Fluor 488-Phalloidin (Molecular Probes, OR) were incubated in immunofluorescence staining buffer (PBS + 5% FBS (Gibco, MD) + 0.1% Triton X-100).

For TRAP staining reagents were purchased from Cosmo Bio Co, CA and used according to instructions of the manufacturer. Cells were stained for ~45 min with chromogenic substrate.

Hoechst and Phalloidin staining fluorescent microscopy images were collected using DAPI, GFP, and Phase Contrast filter sets (BioTek, VT). For TRAP Staining Color brightfield images of the resulting staining were collected. Both, images were collected using a Lionheart FX microscope (BioTek, VT) using a 20×/0.45 NA Plan Fluorite WD objective and Gen3.20 software.

#### TRAP Activity

Differentiated osteoclasts were collected by using trypsin (Corning, NY). A total of 30,000 osteoclasts were plated in an OsteoAssay™ Human Bone Plate (Lonza, CA) and cultured for 72 h at 37°C and 5% CO_2_. The media alone was used as background control. TRAP activity was measured in the supernatants using a TRAP solution containing a synthetic substrate (0.1 M sodium acetate (pH = 5.8), 1 mM ascorbic acid. 0.15 M KCL, 10 mM 4-Nitrophenyl Phosphate). Approximately 20 μl aliquot of culture supernatant was mixed with 80 μl TRAP solution and incubated at 37°C for 1 h. The reaction was stopped by addition 100 μl 0.3 N of NaOH. The absorbance was measured at 405 nm in a microplate reader SpectraMax iD3 (Molecular Devices, CA). The background absorbance was subtracted and the values were plotted and used to represent the activity of TRAP.

#### Expansion of Autologous T Cells

T cells were isolated from the non-adherent fraction of the osteoclast differentiation cultures and activated with CD3/CD28 mAbs coated beads (T cell TransAct™, Miltenyi Biotech, Auburn, CA). After 3 days of stimulation, IL-2 (50 U/ml, TECIN™, National Cancer Institute, Frederick, MD) was added to the media. At day 6, the phenotype of the expanded T cells was analyzed by flow cytometry. Prior to surface staining, T cells were stained with LIVE/DEAD staining (Invitrogen, MA), followed by incubation with 1 μg/ml human IgG (Sigma, MO) to block Fc receptors. Cell surface staining were performed using a cocktail of mAbs: CD3 (clone UCHT1) and CD8 (clone RPA-T8) both from BD Biosciences, CA. Cells were acquired using a BD FACS Symphony flow cytometer and analyzed using FlowJo.

#### Coculture T Cell–Osteoclast (T Cell:OCs)

T cells (100,000 cells) were cultured alone and in the presence of autologous osteoclasts (20,000 cells) at a ratio 5:1 in 96 well plates. Cells were stimulated with CD3/CD28 mAbs coated beads (Miltenyi Biotech, CA) and media as control. Cultures were performed in the presence or absence of IL-27 (50 ng/ml, PeproTech, NJ). Cells were cultured overnight at 37°C and 5% CO_2_ and the supernatants were collected to the analysis of cytokines IFNγ, TNFα, IL-10, IL-17A, and RANKL (LegendPlex™, Biolegend, CA).

For detection of IL-10 secreted by OCs, OCs cultured alone were *in vitro* stimulated with R848 (10 μM, *In vivo*gen, CA) overnight and IL-10 measured in the supernatant (LegendPlex™, Biolegend, CA).

#### Flow Cytometry

T cells were stimulated with CD3/CD28 mAbs overnight and stained with an IL-10 Secretion Assay-Detection Kit (Miltenyi Biotech, CA) or surface expression RANKL (clone MIH24, Biolegend, CA). Cells were acquired using a BD FACS Symphony flow cytometer and analyzed using FlowJo.

### Statistical Analysis

Statistical analysis was performed by GraphPad prism software. One-way ANOVA Friedman test and *post hoc* tests non-parametric Wilcoxon test for comparisons between the culture conditions and Mann–Whitney test for comparisons between study groups were used. Bonferroni test was used to adjust for multiple comparisons.

Correlations were performed using nonparametric Spearman correlation and *p*-value ≤0.01 was considered significant.

## Results

### T Cells Cocultured With Osteoclasts Show Enhanced Cytokine Secretion Upon TCR Stimulation

Osteoclasts (OCs) interaction with T cells leads to activation and subsequently the T-cell derived cytokines can influence OCs activity (36, 44–47). In this study, we hypothesized that in the setting of HIV infection, T cell immune activation alters the network of cytokines involved in the T cell–OCs crosstalk. To address this question, we developed an *in vitro* coculture system of activated T cells and *in vitro* differentiated autologous osteoclasts (OCs) from PBMCs to study their impact in T cell secretion of cytokines. We evaluated cytokines that has been shown to be “pro-osteoclastic”, TNFα, IL-17, and IFNγ; and IL-10 which modulates T cell function and have inhibitory properties in OCs (44–46, 48–52).

PBMCs from PWH (n = 26, red and blue symbols) have median CD4 counts of 524.5 (IQR: 442.3–678.5) cells/μl. The characteristics of the study groups is shown in **Table 1**. A set of participants (n = 17, **Table 1**, HIV^+^ (T), blue symbols) have measurements of bone mineral density (BMD). This group have a median age of 48 (IQR 42–53) years, median CD4 and CD8 T cell counts 525 (IQR: 449.5–689.0) and 718 (IQR: 571.0–1227.0) cells/μl respectively (**Table 1**, HIV^+^ (T)). PBMCs from healthy volunteers (n = 10, back symbols) have a median age 53 (IQR: 38 to 60) years were used as controls (**Table 2**).

Total T lymphocytes from healthy controls and PWH groups were expanded by TCR stimulation with CD3/CD28 mAbs. The frequency of CD4 and CD8 T cells from healthy controls were 67.6% (IQR: 34.85–73.25) and 27.80% (23.25–60.8) respectively (**Figure S1A**). In PWH, the frequency of CD4 and CD8 T cells were 44% (IQR: 23.8–61.85) and 51% (IQR: 29.5–68.95) respectively in PWH (**Figure S1A**). The frequency of expanded CD4 and CD8 T cells was not different between the groups (**Figure S1A**).

*In vitro* differentiation of OCs was monitored by microscopy for the formation of multinucleated osteoclasts by staining the actin cytoskeleton (Phallodin) and nuclei (Hoechst) before and after differentiation, and chromogenic staining for TRAP (**Figures S1B, C**). In addition, TRAP activity was measured in the supernatants of OCs cultured in bone plates (**Figure S1D**).

**Figure 1:**
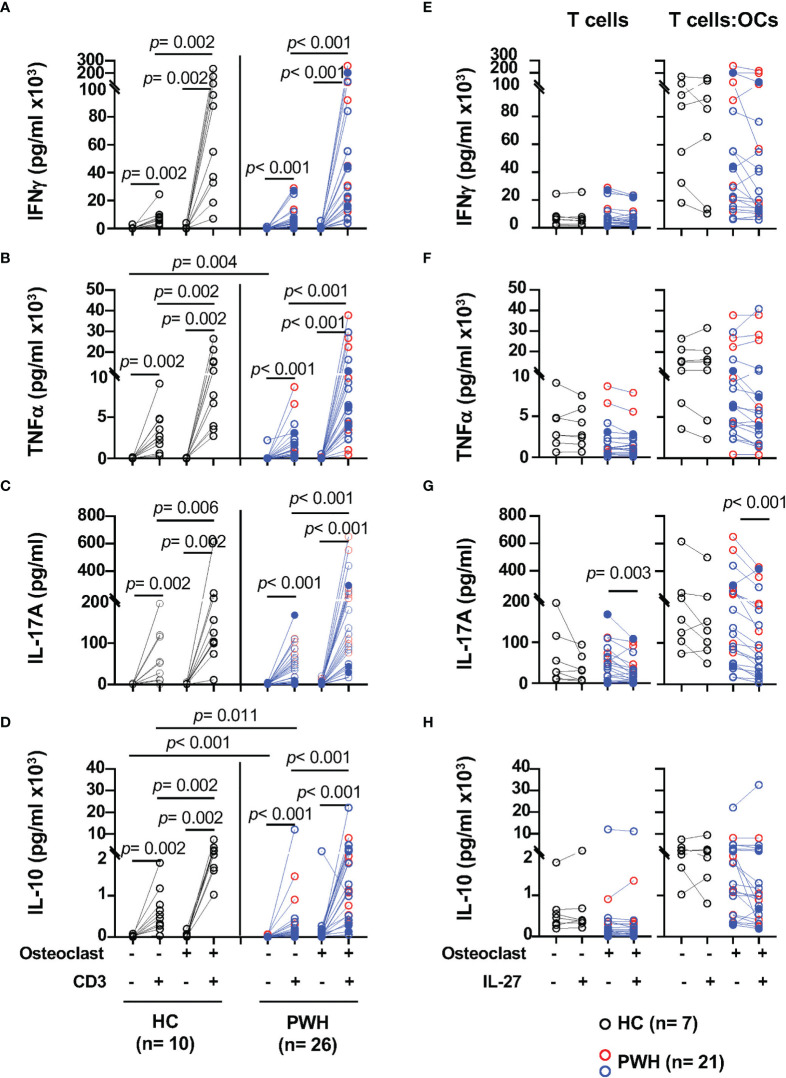
T cells cocultured with osteoclasts (OCs) show enhanced secretion of IFNγ, TNFα, IL-17, and IL-10 upon TCR stimulation. Activated T cells from PWH (n = 9, red symbols, HIV^+^ (T) n = 17, opened blue symbols, **Table 1**) and healthy controls (HC, n = 10, black symbols) were cultured alone or in presence of *in vitro* differentiated autologous osteoclasts at a 5:1 ratio (T cell:OCs). T cells cultured in media were stimulated with CD3/CD28 mAbs overnight and the supernatants were collected to measure cytokines: **(A)** IFNγ, **(B)** TNFα, **(C)** IL-17, and **(D)** IL-10. In the graph, solid blue symbols represent individuals with viral loads >50 copies/ml. T cells cultured alone or in presence of OCs from PWH (n = 7, red symbols, HIV^+^ (T) n = 14, opened blue symbols, **Table 1**) and healthy controls (HC, n = 7, black symbols) were stimulated with CD3/CD28 mAbs in the presence and absence of IL-27 (50 ng/ml) stimulated T cells overnight and the supernatants were collected to measure cytokines: **(E)** IFNγ, **(F)** TNFα, **(G)** IL-17, and **(H)** IL-10. One-way ANOVA was used for comparisons between culture conditions. *Post hoc* non-parametric Wilcoxon was used for comparisons between culture conditions. Bonferroni test was used to adjust for multiple comparisons. *P*-value ≤0.003 was considered significant. *Post hoc* nonparametric unpaired Mann–Whitney test was used for comparisons between the groups adjusted by Bonferroni test. *P*-value ≤0.01 was considered significant.

Activated T cells from PWH (n = 26) and healthy controls (n = 10) were cultured alone or in presence of OCs at 5:1 ratio (T cells:OCs) and stimulated with CD3/CD28 mAbs. After 24 h of culture, IFNγ, TNFα, IL-17, and IL-10 were measured in the supernatants (**Figure 1**). Unstimulated T cells cultured alone or in presence of OCs showed low levels of cytokine secretion in both healthy controls and PWH groups (**Figures 1A-D** **)**. Similarly, unstimulated OCs cultured alone showed low basal level of cytokines secretion in the supernatants (**Figure S2A**).

TCR stimulation of T cells from healthy controls significantly increases secretion of IFNγ (*p* = 0.002), and it was enhanced by the presence of OCs in the cultures (*p* = 0.002) (**Figure 1A**). Similar effects were observed upon stimulation of T cells from PWH (*p <*0.001) when compared to T cells cultured alone (**Figure 1A**). The levels of IFNγ in the supernatants of the T cell cultures in the absence or the presence of OCs were similar between healthy controls and PWH (**Figure 1A**).

In addition, OCs also significantly enhanced the secretion of TNFα in healthy controls and PWH, *p* = 0.002 and *p* < 0.001 respectively (**Figure 1B**). Compared to stimulated T cells cultured alone, the ability of T cells to secrete IL-17A was also significantly enhanced by the presence of OCs in the cocultures in both PWH (*p <*0.001) and healthy controls (*p* = 0.006) (**Figure 1C** **)**.

We next evaluated the secretion of the immunomodulatory cytokine IL-10 and asked whether OCs can modulate its secretion (47, 49–52). Stimulation of T cells in the presence of OCs significantly induced secretion of IL-10 relative to T cells cultured alone in PWH and healthy controls *p <*0.001 and *p* = 0.002 respectively (**Figure 1D**). T cells from PWH expressed lower basal levels of IL-10 and a trend was observed after stimulation of T cells cultured alone but did not reach statistical significance (**Figure 1D**).

These results suggest that OCs enhance the secretion of Th1 (IFNγ, TNFα) and Th17 (IL-17) associated cytokines in both healthy control and PWH groups. In addition to the enhancement of pro-inflammatory cytokines, OCs also promoted secretion of IL-10 in the T cell:OCs cocultures suggesting a complex interplay between OCs and T cells.

### IL-27 Modulation of IL-17 Secretion During T Cell–Osteoclasts Interaction

The above data suggest that OCs enhance activation of T cells promoting secretion of pro- and anti-inflammatory cytokines in the bone (33). IL-27 is an immune modulatory cytokine that inhibit OCs differentiation (76). In T cells, IL-27 promotes Th1 differentiation, suppresses Th17 differentiation and induces secretion of IL-10 by regulatory T cells (46, 49–57). Whether IL-27 can exert these functions in the setting of HIV infection is not well defined. We next evaluated the effects of IL-27 when T cells were TCR stimulated alone or in the presence of OCs (**Figure 1**, right panels).

IL-27 showed no effect on IFNγ and TNFα secretion by T cells from both study groups (**Figures 1E, F**). In contrast, IL-27 induced a significantly downregulation in IL-17 secretion in PWH but not in healthy volunteers (**Figure 1G**).

The secretion of IL-10 showed a trend of inhibition by IL-27, however did not reach statistical significance (**Figure 1H**). Because both T cells and OCs can secrete IL-10, to better understand their relative contribution and its regulation by IL-27 we examined the secretion of IL-10 in both cell types (47). T cells were evaluated using a flow cytometric cytokine capture assay in a set of HIV infected individuals (n = 10, **Figures S2B, C**). We found that both CD4 and CD8 T cells produce IL-10, and the frequency of IL-10^+^CD4 and IL-10^+^CD8 T cells was significantly increased in the cocultures with OCs, however IL-27 did not have an effect in either the frequency or median fluorescence intensity of IL-10 secreting T cells (**Figures S2B, C**). In addition, OCs cultured alone secrete low basal levels of IL-10 that was significantly increased upon stimulation with the TLR7/8 agonist in both healthy controls and PWH. In this culture condition, IL-27 showed no effect on IL-10 secretion (**Figure S2D**).

Altogether these data suggest that IL-27 may not modulate IFNγ, TNFα and IL-10 in activated T cells. In contrast in PWH, IL-27 showed downregulatory effect on IL-17 secretion and overcame the costimulatory effects of OCs in the cocultures.

### IL-27 Modulates RANKL Expression in CD4 T Cells From PWH

**Figure 2:**
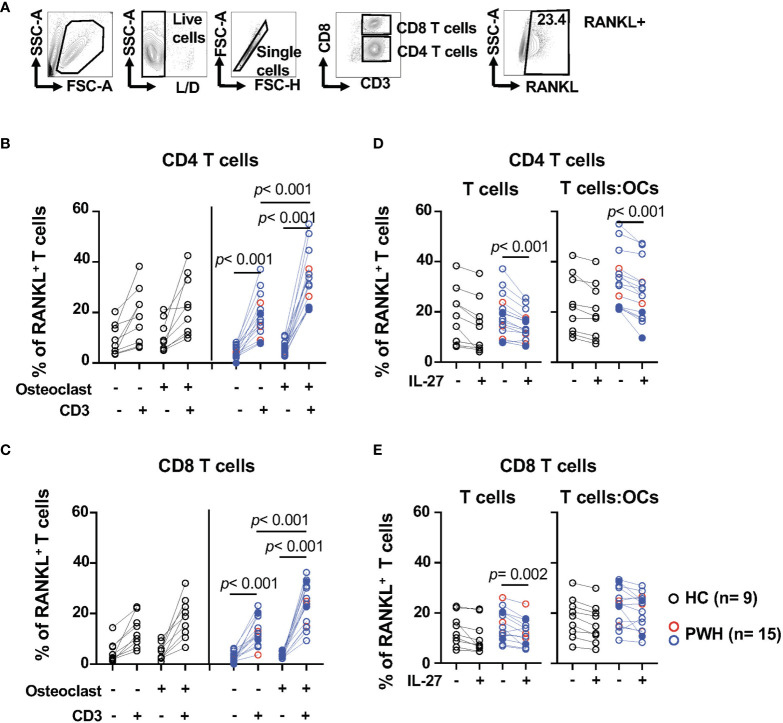
IL-27 modulates the expression surface expression of RANKL in CD4 T cells from PWH. T cells from PWH (n = 3, red symbols, HIV^+^ (T) n = 12, opened blue symbols, **Table 1**) and healthy controls (HC, n = 9, black symbols) were cultured alone or in presence of OCs at a 5:1 ratio (T cell:OCs) were stimulated with CD3/CD28 mAbs. After overnight culture RANKL was measured by flow cytometry. **(A)** Gating strategy and representative contour plot of RANKL surface expression in T cells, **(B)** Surface expression of RANKL in CD4 T cells, and **(C)** Surface expression of RANKL in CD8 T cells. T cells cultured alone or in presence of OCs were stimulated with CD3/CD28 mAbs in the presence and absence of IL-27 (50 ng/ml) and RANKL surface expression was measure by flow cytometry: **(D)** CD4 T cells and **(E)** CD8 T cells. Graph representing RANKL surface expression in T cells. In the graph, solid blue symbols represent individuals with viral loads >50 copies/ml. One-way ANOVA was used for comparisons between culture conditions. *Post hoc* non-parametric Wilcoxon was used for comparisons between culture conditions. Bonferroni test was used to adjust for multiple comparisons. *P*-value ≤0.003 was considered significant. *Post hoc* nonparametric unpaired Mann–Whitney test was used for comparisons between the groups adjusted by Bonferroni test. *P*-value ≤0.01 was considered significant.

The data above suggest that IL-27 can modulate IL-17, a cytokine that has been shown to synergized with RANKL, a major “pro-osteoclastic” factor secreted by activated T cells (59, 79, 82). To better understand the potential role of IL-27 in the regulation of cytokines in the setting of HIV infection, we evaluated RANKL expression in CD4 and CD8 T cells from PWH (n = 15) and healthy controls (n = 9) by flow cytometry (**Figure 2**). In PWH, both CD4 and CD8 T cells cultured alone showed upregulation of surface expression of RANKL upon TCR stimulation, and only a trend was noted in healthy controls (**Figures 2A-C**). The presence of OCs in the T cell cultures increased the frequency of RANKL^+^CD4 and CD8 T cells from PWH (**Figures 2B, C**). Similar observations were noted in the median fluorescence intensity of RANKL in T cells (**Figures S3A, B**)

We next evaluated the effects of IL-27 on RANKL expression (**Figures 2D, E**). IL-27 showed an inhibitory effect on the frequency of RANKL^+^ T cells cultured alone from PWH (**Figures 2D, E**). IL-27 inhibitory effect was still observed in CD4 T cells stimulated in the presence of OCs (p <0.001, **Figure 2D**) but not in CD8 T cells. These effects were not observed in the median fluorescence intensity (**Figures S3C, D**). The effects of IL-27 on CD4 T cells were not reflected in the levels of RANKL detected in the supernatants (**Figure S3E**).

These data suggest that activated CD4 and CD8 T cells expressed surface RANKL and this expression is significantly enhanced by OCs in PWH. In addition, IL-27 inhibited RANKL expression in CD4 but not CD8 T cells cultured with OCs.

### IFNγ Secretion by T Cells is Associated With Bone Mineral Density in PWH

In the context of HIV infection, systemic inflammation and immune activation has been suggested as contributor of bone loss (83). The data above suggest that T cell function is enhanced by osteoclasts potentiating their ability to secrete pro-inflammatory cytokines, namely, IFNγ, TNFα, IL-17, RANKL, and the anti-inflammatory cytokine IL-10.

**Figure 3:**
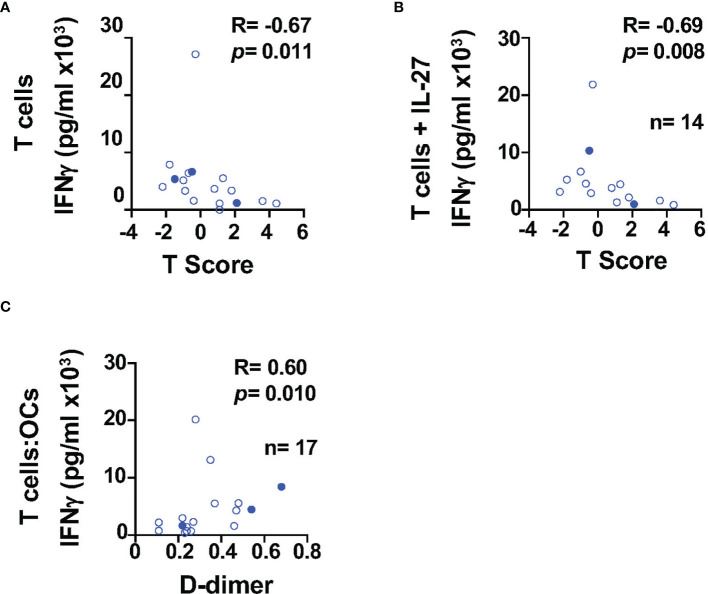
IFNγ secretion is associated with BMD in PWH. Relationship between IFNγ secretion and BMD in T cells cultured: **(A)** alone (n=17), **(B)** in presence of IL-27 (n = 14). **(C)** Relationship between IFNγ secretion in the T cell:OCs cocultures (n = 17) and D-dimer. Correlations were performed using nonparametric Spearman correlation and p-value ≤0.01 was considered significant.

To better understand the impact of HIV infection in the bone, we investigated the relationship between cytokines and BMD (T-score), markers of systemic inflammation and coagulation, namely, CRP (C-reactive protein) and D-dimer (a bioproduct of fibrin degradation). The study participants (n = 17, **Table 1** HIV^+^(T)) had CD4 T cell counts of CD4 of 525.0 (IQR: 449.5–689.0) cells/μl and CD8 T cell counts of 718.0 (571.0–1,227.0) cells/μl. The median T score was −0.3 (IQR: −0.95–1.55), and six out of 17 study participants had metabolic syndrome (**Table 1**). We found a weak trend of an inverse correlation between the T score and the ability of T cells to secrete IFNγ in absence of IL-27 (R = −0.67, *p* = 0.011), (**Figure 3A** **)**. This inverse association was significant when T cells were cultured in the presence of IL-27 (R = −0.69, *p* = 0.008, **Figure 3B**) although the levels of IFNγ were not different between the culture conditions (**Figure 1E**). This association was not observed when the levels of IFNγ secretion increased as a result of the costimulatory function of the OCs (**Figure 1E**). In addition, the levels of IFNγ in the T cell:OCs cocultures showed a weak association with the biomarker of inflammation and coagulation D-dimer (R = 0.60, *p* = 0.010), (**Figure 3C**). No association was observed with CRP plasma levels.

Low bone mineral density has been associated with higher lipid levels (84–88). Because some of the study participants had metabolic syndrome, we evaluated the relationship between lipids and cytokines. Total cholesterol and LDL and showed a positive trend with the levels of TNFα secretion by T cells (R = 0.60, *p* = 0.016 and R = 0.60, *p* = 0.012 respectively) but did not reach statistical significance (**Figure S3F**).

These data suggest that Th1 cytokines (IFNγ and TNFα) in PWH may be a contributing factor in maintaining levels of inflammation and increased risk of bone loss.

## Discussion

In PWH, systemic inflammation and immune activation is associated with comorbidities including bone loss. In the present study, in an *in vitro* coculture system of T cells and OCs from PWH and healthy controls, we evaluated the effect of T cell immune activation on cytokine secretion, and the potential regulatory effect of IL-27. We found that OCs can significantly enhance T cell function promoting the secretion of both pro- (IFNγ, TNFα, IL-17) and anti-inflammatory (IL-10) cytokines in healthy controls and PWH. In addition, the presence of OCs in the cocultures increased T cell surface expression of RANKL, a major pro-osteoclastic factor secreted by T cells. IL-27 showed no regulatory effects on the secretion of IFNγ, TNFα, and IL-10 by activated T cells. In contrast, IL-27 inhibited the secretion of IL-17 and surface RANKL expression in CD4 T cells from PWH. All together these results suggest that in the context of HIV infection IL-27 may favor Th1 associated cytokines (IFNγ and TNFα).

IL-27 has been shown to have direct and indirect effects in bone remodeling. *In vitro*, IL-27 inhibits RANKL dependent OCs differentiation of human monocytes and also inhibits bone resorption suggesting an anti-inflammatory function in bone homeostasis (76, 77, 89). *In vivo*, in a murine model of collagen-induced arthritis, IL-27 induced Th1 differentiation and IFNγ secretion constraining osteoclast differentiation (90–93). In contrast, IFNγ can stimulate osteoclast formation by promoting T cell activation and secretion of pro-osteoclastogenic factors including RANKL and TNFα leading to bone loss in *in vivo* models of bone resorption, namely, ovariectomy, LPS injection, and inflammation (45, 59). Our present study shows that *in vitro* OCs have a costimulatory effect on secretion of cytokines including IFNγ. The negative association of IFNγ levels with T-score may suggest a potential contribution of T cell immune activation. One limitation of this study is that the participants have normal T scores and future studies should further investigate the cytokine secretion networks involved in PWH with low BMD.

IL-27 showed no effects in the regulation of IFNγ in the cocultures. In our recent report we found that IL-27 promotes secretion of IFNγ in TIGIT^+^HIVGag specific T cells by upregulation of T-bet, a transcription factor associated with Th1 differentiation (94). It is possible that in the present studies the effects of IL-27 on IFNγ secretion were not fully appreciated in polyclonally activated T cells from healthy controls and PWH. In addition, recent reports have shown that OCs can be infected by HIV-1 (95, 96). OCs also can act as antigen presenting cells and activate CD4 and CD8 T cells (47). In this scenario, the interaction of T cells and OCs may contribute to maintain T cell immune activation, secretion of pro-osteoclastogenic cytokines and viral dissemination impacting bone homeostasis in PWH.

In contrast to IFNγ and TNFα, IL-27 inhibited IL-17 secretion in T cells cocultured with OCs. Th17 cells also produce RANKL and promote IL-17-dependent osteoclast differentiation by increasing expression of RANK receptor and promoting secretion of RANKL by osteoblasts (82, 97, 98). In our studies we found that IL-27 inhibited RANKL in CD4 T cells suggesting that IL-27 may has an anti-inflammatory effect during T cell interaction with OCs, whether RANKL inhibition was achieved in IFNγ and/or IL-17 secreting T cells needs to be determined.

IL-27 has been suggested to have an anti-inflammatory function; and in our *in vitro* coculture system, we found that in the context of HIV infection favors a Th1 associated cytokines by suppressing IL-17 secretion. The levels of IL-27 during HIV infection seems to be unmodulated; similar plasma levels have been reported in untreated, and successfully suppressed viremia with cART PWH, and healthy controls (99). In contrast, two small studies reported contradictory changes of plasma levels of IL-27 during HIV infection (100, 101).

In addition, to its immunomodulatory functions, *in vitro* IL-27 has antiviral properties, and inhibits HIV replication in CD4 T cells, monocyte-derived macrophages, and dendritic cells (102–104). It has been determined that OCs can be infected by HIV promoting their differentiation, whether IL-27 can protect OCs from HIV infection is largely unknown (95, 96).

Future studies should evaluate the expression of IL-27 in the bone and its potential overall impact on OCs activity at the sites of bone remodeling in PWH.

## Data Availability Statement

The original contributions presented in the study are included in the article/**Supplementary Material**. Further inquiries can be directed to the corresponding author.

## Ethics Statement

The studies involving human participants were reviewed and approved by the MedStar Georgetown University Hospital and a NIAID Institutional Review Board. The patients/participants provided their written informed consent to participate in this study.

## Author Contributions

MC designed the study. TL performed the experiments. MC and TL analyzed and interpreted the data and write the manuscript. CH, JK, and PK were involved in recruitment of participants of the study and write the manuscript. JMW stained and imaged osteoclasts. JQ contributed with the statistical analysis. All authors listed have made a substantial, direct, and intellectual contribution to the work and approved it for publication.

## Funding

This work supported by the Leidos Biomedical Research, Inc. has been funded in whole or in part with federal funds from the National Cancer Institute, NIH, under Contract HHSN261200800001E. The content of this publication does not necessarily reflect the views or policies of the Department of Health and Human Services, nor does mention of trade names, commercial products, or organizations imply endorsement by the U.S. Government. The funder was not involved in the study design, collection, analysis, interpretation of data, the writing of this article or the decision to submit it for publication. MC is also supported in part by the National Institute of Allergy and Infectious Diseases of the National Institutes of Health under award number NIH R01AI145549-02 and by the District of Columbia Center for AIDS Research, an NIH funded program (P30AI117970) which is supported by the following NIH Co-Funding and Participating Institutes and Centers: NIAID, NCI, NICHD, NHLBI, NIDA, NIMH, NIA, NIDDK, NIMHD, NIDCR, NINR, FIC and OAR. TGM is supported by the Intramural Research Program of the NIH. The work of JW was supported by the Intramural Research Program of the Eunice Kennedy Shriver National Institute of Child Health and Human Development, National Institutes of Health (ZIA Act HD001501 to L.V. Chernomordik).

## Conflict of Interest

The authors declare that the research was conducted in the absence of any commercial or financial relationships that could be construed as a potential conflict of interest.

## Publisher’s Note

All claims expressed in this article are solely those of the authors and do not necessarily represent those of their affiliated organizations, or those of the publisher, the editors and the reviewers. Any product that may be evaluated in this article, or claim that may be made by its manufacturer, is not guaranteed or endorsed by the publisher.
