# Supplementary material for: IL-27 Modulates the Cytokine Secretion in the T Cell–Osteoclast Crosstalk During HIV Infection
Source: Front Immunol. 2022 Apr 5;13:818677. doi: 10.3389/fimmu.2022.818677 (PMC9037094; doi:10.3389/fimmu.2022.818677)
Supplement: Supplementary file 1 [file DataSheet_1.pdf]

Supplemental Materials

Figure S1. In vitro T cell expansion and osteoclasts differentiation.

(A) T cells were isolated from osteoclast differentiation cultures and *in vitro* stimulated with CD3 and CD28 mAbs (coated beads) in the presence of IL-2 for 6 days. The frequency of CD4 and CD8 T cells from PWH (n= 25) and healthy controls (n= 9) was measured by flow cytometry. Osteoclasts were differentiated from frozen PBMCs from PWH (n= 26) and healthy controls (n= 10) in conditioned media containing (M-CSF and RAKL) as described in material and methods. **(B)** The formation of multinucleated osteoclasts was confirmed via florescent microscopy by staining the actin cytoskeleton (Phallodin) and nuclei (Hoechst) before and after differentiation and by **(C)** chromogenic staining for TRAP (representative images)**. (D)** Osteoclasts differentiation was assessed in PWH (n= 23) and healthy controls (n= 10) by culturing the osteoclasts in OsteoAssay^{TM} Human Bone Plate for 72 hours. TRAP activity was measured in the supernatants using a synthetic substrate p-Nitrophenylphosphate as described in material and methods. Mann-Whitney test was used for comparisons between the groups. *P* value ≤ 0.05 was considered significant.

Figure S2. IL-10 is secreted by T cells in the cocultures with osteoclasts.

(A) *In vitro* differentiated osteoclasts from HC (n= 10) and PWH (n= 19) were cultured alone with media overnight. The secretion of IFNγ, TNFα, IL-17, and IL-10 by osteoclasts cultured alone was measured in supernatant. In the graph, solid blue symbols represent individuals with viral loads > 50 copies/mL. T cells from PWH (HIV$^+$ (T) n= 10) cultured overnight in the presence or absence of osteoclasts were stained with IL-10 Secretion Assay-Detection Kit. **(B)** Gating Strategy. **(C)** The expression of IL-10 expressed as frequency and Median Flourescence

Intensity (MFI) of IL-10^{+}CD4 and IL-10^{+}CD8 T cells. In the graphs, solid blue symbols represent individuals with viral loads > 50 copies/mL. **(D)** *In vitro* differentiated osteoclasts from HC (n= 7) and PWH (n= 16) were stimulated with R848 (10 μM) in the presence or absence of IL-27. After overnight culture, IL-10 secretion was measured in supernatant. The graph is represented by box and whisker showing the median value with first and third quartiles in the box, with whiskers extending to the minimum and maximum values. One-way ANOVA was used for comparisons between culture conditions. Post hoc non- parametric Wilcoxon was used for comparisons between culture conditions with Bonferroni adjustment. *P* value ≤ 0.02 was considered significant for comparison between three culture conditions. Post hoc nonparametric unpaired Mann-Whitney test was used for comparisons between the groups adjusted by Bonferroni test. *P* value ≤ 0.01 was considered significant.

Figure S3. IL-27 modulate the expression surface expression of RANKL in CD4 T cells from PWH. T cells from PWH (n=3, red symbols, HIV^{+} (T) n=12, opened blue symbols, Table 1) and healthy controls (HC, n= 9, black symbols) were cultured alone or in presence of OCs at a 5:1 ratio (T cell:OCs) were stimulated with CD3/CD28 mAbs. After overnight culture RANKL was measured by flow cytometry. **(A)** Median fluorescence intensity (MFI) of RANKL in CD4 T cells. **(B)** Surface expression of RANKL in CD8 T cells. T cells cultured alone or in presence of OCs were stimulated with CD3/CD28 mAbs in the presence and absence of IL-27 (50 ng/ml) and MFI of RANKL was measure by flow cytometry in **(C)** CD4 T cells and **(D)** CD8 T cells. Graph representing MFI of EANKL in T cells. **(E)** The RANKL secretion in the supernatant was measured. In the graph, solid blue symbols represent individuals with viral loads > 50

copies/mL. **(F)** Relationship between TNFα secretion of T cells cultured alone and total cholesterol and LDL levels.

One-way ANOVA was used for comparisons between culture conditions. Post hoc non-parametric Wilcoxon was used for comparisons between culture conditions with Bonferroni adjustment. P value ≤ 0.003 was considered significant. Post hoc nonparametric unpaired Mann-Whitney test was used for comparisons between the groups adjusted by Bonferroni test. P value ≤ 0.01 was considered significant. Correlations were performed using nonparametric Spearman correlation and p value ≤ 0.01was considered significant.

Figure S1

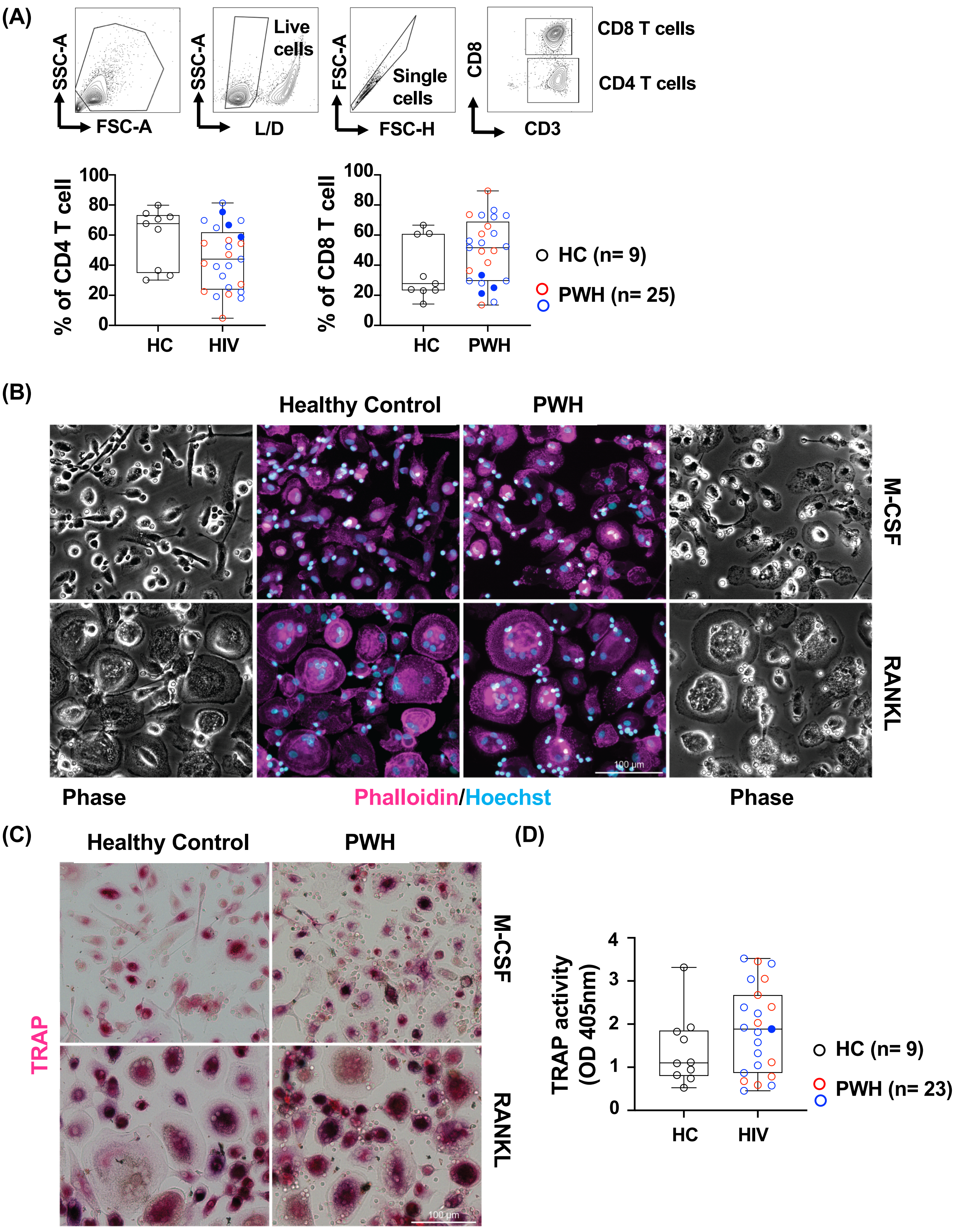
(A)
SSC-A
FSC-A
SSC-A
L/D
Live cells
FSC-A
FSC-H
Single cells
CD8
CD3
CD8 T cells
CD4 T cells
% of CD4 T cell
100
80
60
40
20
0
HC
HIV
% of CD8 T cell
HC
PWH
HC (n= 9)
PWH (n= 25)
(B)
Healthy Control
PWH
M-CSF
RANKL
100 µm
Phase
Phalloidin/Hoechst
Phase
(C)
Healthy Control
PWH
TRAP
M-CSF
RANKL
100 µm
(D)
TRAP activity (OD 405nm)
4
3
2
1
0
HC
HIV
HC (n= 9)
PWH (n= 23)

Figure S2

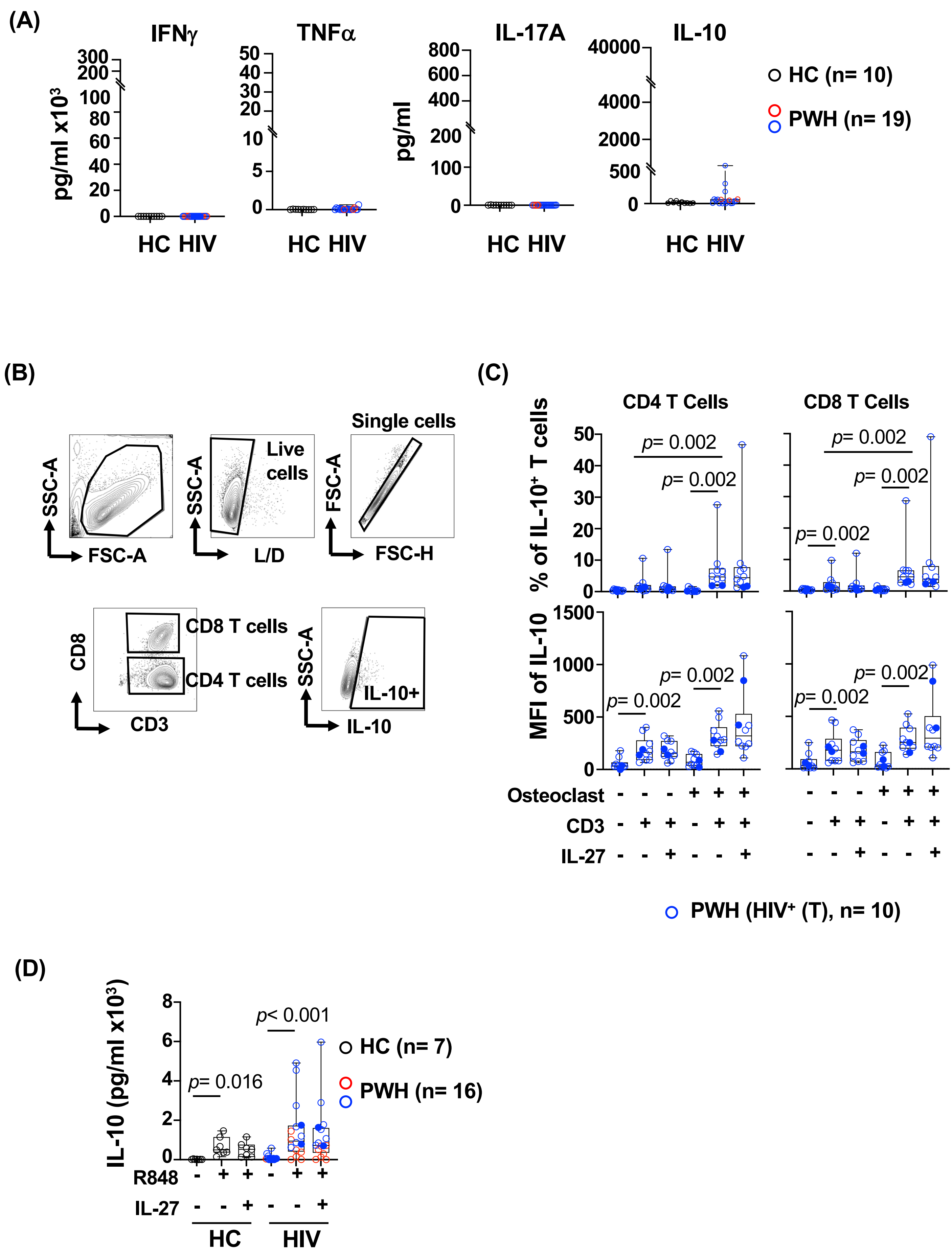

(A)
IFNγ
TNFα
IL-17A
IL-10
pg/ml x10³
pg/ml
HC HIV
HC (n= 10)
PWH (n= 19)
(B)
SSC-A
FSC-A
Live cells
L/D
Single cells
FSC-A
FSC-H
CD8 T cells
CD4 T cells
CD8
CD3
SSC-A
IL-10+
IL-10
(C)
CD4 T Cells
CD8 T Cells
% of IL-10⁺ T cells
MFI of IL-10
p= 0.002
Osteoclast
CD3
IL-27
PWH (HIV⁺ (T), n= 10)
(D)
IL-10 (pg/ml x10³)
p= 0.016
p< 0.001
R848
IL-27
HC
HIV
HC (n= 7)
PWH (n= 16)

Figure S3

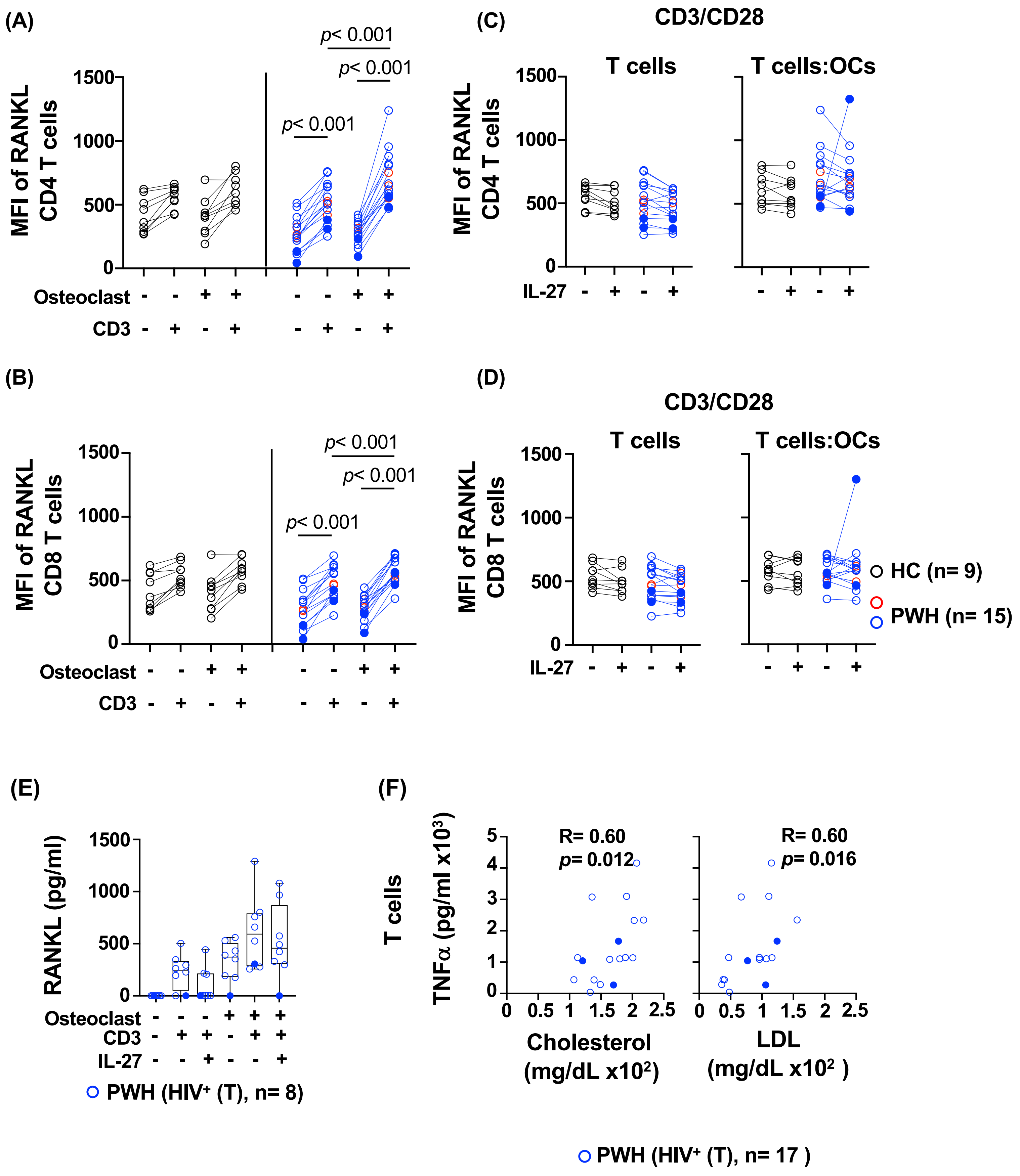
(A)
MFI of RANKL CD4 T cells
1500
1000
500
0
p< 0.001
p< 0.001
p< 0.001
Osteoclast - - + + - - + +
CD3 - + - + - + - +
(B)
MFI of RANKL CD8 T cells
1500
1000
500
0
p< 0.001
p< 0.001
p< 0.001
Osteoclast - - + + - - + +
CD3 - + - + - + - +
(C)
CD3/CD28
T cells
T cells:OCs
MFI of RANKL CD4 T cells
1500
1000
500
0
IL-27 - + - + - + - +
(D)
CD3/CD28
T cells
T cells:OCs
MFI of RANKL CD8 T cells
1500
1000
500
0
IL-27 - + - + - + - +
HC (n= 9)
PWH (n= 15)
(E)
RANKL (pg/ml)
1500
1000
500
0
Osteoclast - - - + + +
CD3 - + + - + +
IL-27 - - + - - +
PWH (HIV+ (T), n= 8)
(F)
T cells
TNFα (pg/ml x10³)
5
4
3
2
1
0
R= 0.60
p= 0.012
R= 0.60
p= 0.016
0 0.5 1 1.5 2 2.5
Cholesterol (mg/dL x10²)
LDL (mg/dL x10²)
PWH (HIV+ (T), n= 17)
